# Extracellular vesicles lay the ground for neuronal plasticity by restoring mitochondrial function, cell metabolism and immune balance

**DOI:** 10.1177/0271678X251325039

**Published:** 2025-03-12

**Authors:** Dirk M Hermann, Chen Wang, Ayan Mohamud Yusuf, Josephine Herz, Thorsten R Doeppner, Bernd Giebel

**Affiliations:** 1 Department of Neurology, 39081 University Hospital Essen, University of Duisburg-Essen, Essen, Germany; 2 Department of Pediatrics I, 39081 University Hospital Essen, University of Duisburg-Essen, Essen, Germany; 3 Department of Neurology, University Hospital Gießen and Marburg, Justus-Liebig-University Gießen, Gießen, Germany; 4 Institute for Transfusion Medicine, 39081 University Hospital Essen, University of Duisburg-Essen, Essen, Germany

**Keywords:** Anti-inflammation, energy metabolism, exosome, immune modulation, mitochondria, oxidative stress, synaptic plasticity

## Abstract

Extracellular vesicles (EVs) convey complex signals between cells that can be used to promote neuronal plasticity and neurological recovery in brain disease models. These EV signals are multimodal and context-dependent, making them unique therapeutic principles. This review analyzes how EVs released from various cell sources control neuronal metabolic function, neuronal survival and plasticity. Preferential sites of EV communication in the brain are interfaces between pre- and postsynaptic neurons at synapses, between astrocytes and neurons at plasma membranes or tripartite synapses, between oligodendrocytes and neurons at axons, between microglial cells/macrophages and neurons, and between cerebral microvascular cells and neurons. At each of these interfaces, EVs support mitochondrial function and cell metabolism under physiological conditions and orchestrate neuronal survival and plasticity in response to brain injury. In the injured brain, the promotion of neuronal survival and plasticity by EVs is tightly linked with EV actions on mitochondrial function, cell metabolism, oxidative stress and immune responses. Via the stabilization of cell metabolism and immune balance, neuronal plasticity responses are activated and functional neurological recovery is induced. As such, EV lay the ground for neuronal plasticity.

## Introduction

The damage to neurons, axons, or synapses in response to various neurological diseases results in neuronal network disruption.^1,2^ The release of danger-associated molecular patterns (DAMPs) induces a pro-inflammatory tissue microenvironment associated with oxidative stress,^3,4^ which may critically exacerbate pre-existing mitochondrial disturbances^5,6^ and may propagate tissue damage to distant brain areas^7^ or even outside the brain.^3^ Glial and vascular cells are intricately involved in this pro-inflammatory response and may deteriorate mitochondrial respiration failure in neurons via mechanisms involving metabolic reprogramming and oxidative stress.^8,9^ The complexity of brain damage under conditions such as stroke or traumatic brain injury creates the need of large-scale brain tissue remodeling to compensate for lost functions. Unfortunately, the regenerative potential of the injured brain is limited, which is partly due to age^10,11^ and vascular risk factors, such as dyslipidemia and diabetes,^12–15^ which may exacerbate mitochondrial dysfunction,^16^ thus compromising neuronal survival and plasticity.^1^ Persistent neurological deficits associated with daily life impairments impose huge health burdens.^17^

Extracellular vesicles (EVs) play a crucial role in cellular responses to brain injuries and decisively control neuronal survival and plasticity.^18,19^ EVs are cell-derived, lipid membrane-enclosed vesicles carrying a broad spectrum of biologically active signaling molecules (most notably proteins, RNAs and bioactive lipids).^20^ Dependent on the tissue origin of the producer cells and the molecular determinants of the recipient cells,^21^ EVs can constrain inflammatory responses, reduce neuronal injury, and promote neuronal plasticity.^18,22^ EVs can also carry energy-rich substrates, enzymes and respiratory chain components that restore energy metabolism, thus enhancing brain recovery.^23,24^ In addition, EVs have been shown to transfer membrane fragments and entire cell organelles, including mitochondria, to target cells.^25,26^ In contrast to pharmacological compounds, which act by modulating defined signaling pathways, EVs possess multiple abilities^18^ allowing the modulation of complex disease processes in a highly synergistic and context-dependent way.^20,22^

When delivered therapeutically in animal disease models, stem/progenitor cell-derived EVs of different origins can exhibit striking neurological recovery-promoting activities.^19^ In the middle cerebral artery occlusion (MCAO) model, for example, intravenously administered small mesenchymal stromal cell (MSC)-derived EVs within the size of exosomes enhanced motor-coordination recovery similar to parental MSCs by mechanisms involving long-term neuroprotection, neurogenesis, axonal sprouting, remyelination, and synaptic plasticity.^27,28^ In the neonatal hypoxia-ischemia model, intranasally delivered small MSC-EVs improved long-term neurodevelopmental outcome associated with increased angiogenesis and restored myelination.^29^ In transgenic Alzheimer’s disease models, intranasally administered small MSC-EVs reduced the progression of cognitive deficits via mechanisms involving the polarization of microglia to an anti-inflammatory phenotype and reduction of cerebral β-amyloid (Aβ) plaque load.^30,31^ In view of these potent actions, this review aims to outline our current understanding of the mechanisms of action of EVs, describing how EVs from various cellular sources regulate neuronal mitochondrial function, cell metabolism and plasticity in the healthy and the injured brain. The joint evidence of these studies indicates that neuroprotective effects of MSC-EVs are conserved across different maturation phases of the brain, and they are still evident under neurodegenerative conditions.

In preparation of this review, we performed a detailed literature search in Pubmed combining the keywords (“extracellular vesicle” or “exosome”) and (“neuron” or “neuronal survival” or “neuroprotection” or “neuronal plasticity” or “axonal plasticity” or “synaptic plasticity” or “neurological recovery”) and (“mitochondrial” or “energy state” or “metabolism” or “metabolic” or “oxidative stress” or “inflammation” or “inflammatory”). Besides, a literature search combining the key words (“extracellular vesicle” or “exosome”) and (“ischemic stroke” or “focal cerebral ischemia” or “hypoxia-ischemia” or “traumatic brain injury” or “brain trauma” or “brain injury” or “neurodegeneration” or “neurodegenerative” or “Alzheimer”) and (“mitochondrial” or “energy state” or “metabolism” or “metabolic” or “oxidative stress” or “inflammation” or “inflammatory”) was performed.

## Roles of EVs in intercellular communication

### EV origins and composition

EVs are released by virtually all cells.^22^ They are abundant in all tissues and body liquids, including the brain, CSF and blood. Based on their biogenesis in different cell compartments,^20^ EVs are classified into different categories.

Exosomes, which typically have a size of 60 150 nm, are formed by inward budding of the limiting membrane of late endosomes or autophagosomes/lysosomes (Table 1). Late endosomal and autophagosomal/lysosomal vesicles are released into the extracellular space by plasma membrane fusion.^20^ Based on their GM1 ganglioside content, late endosomal exosomes can be captured by cholera toxin b subunit.^32^ Exosomes formed by late endosomes have important roles in intercellular communication. At the same time, exosomes, specifically those formed by autophagosomes/lysosomes, have roles in cellular waste excretion.^33^

**Table 1.** EV categories defined by cellular biogenesis and their major cargos.

EV category	Cellular biogenesis	Typical size	Major cargos
Late endosomal exosomes	Formed by inward budding of the limiting membrane of late endosomes. Resulting intraluminal vesicles released into the extracellular space by endosomal plasma membrane fusion^20,22^	60–150 nm	Rich in proteins and lipids (including phosphatidic acid, phosphatidylserine, sphingolipids). Probably low RNA content.^32,180^ Surface proteins organized by tetraspanins including CD9, CD63 and CD81^51^, which coordinate cell adhesion via integrin, selectin and cell adhesion molecule clustering. Important roles in cell communication in acute and post-acute injury phases
Autophagosomal/ lysosomal exosomes	Formed by inward limiting membrane budding within autophagosomes/ lysosomes. Resulting intraluminal vesicles released by autophagosomal/lysosomal plasma membrane fusion^20^	60–150 nm, larger EVs exist that may include organelles or organelle fractions, including mitochondria/ mitochondrial fractions (see below)^26^	Cellular waste products (degraded proteins, RNAs and oxidated lipids). Waste excretion mechanism activated, when autophagy is overchallenged or inhibited.^33^ Many contents not involved in cell communication. Degradation/ elimination by target cells
EVs originating from endoplasmic reticulum (ER)	Formed by budding at specific ER membrane contact sites.^36^ Via direct ER-endosomal or ER-lysosomal/autophagosomal contacts, newly-formed proteins are transferred to late endosomes (proteins passing cellular quality control) or lysosomes/autophagosomes (proteins not passing cellular quality control),^37^ from where they are further processed and released	60–150 nm, larger EVs may include organelles or organelle fragments	Rich in RNAs including miRNAs and lncRNAs. Rich in newly-formed proteins that are released for cellular communication (proteins that passed cellular quality control) or cellular waste excretion (proteins that did not pass cellular quality control)
Nuclear EVs	Formed by membrane budding at the inner nuclear membrane.^34,35^ Passaged across the cytosol and released into the extracellular space	60–150 nm	Rich in RNAs, specifically pre-miRNAs.^32^ Pre-miRNAs need to be processed to miRNAs to exert cellular communication roles
Mitochondria or mitochondria fractions	Released as free mitochondria/ mitochondria fractions or EV-encapsulated mitochondria/ mitochondria fractions upon inflammation or injury^26^	60 nm and larger (wide size spectrum)	Structurally and functionally intact or damaged mitochondrial constituents that include proteins, lipids and DNA. Structurally and functionally intact mitochondria/ mitochondrial fractions can restore mitochondrial in injured target cells.^26,38,39^ Structurally and functionally damaged mitochondria/ mitochondrial fractions taken up for cellular degradation/ elimination^40,41^
Microvesicles	Formed by outward budding of the plasma membrane into the extracellular space^20,22^	100–1000 nm	Can traffic DAMPs, including IL1α, IL1β and RANTES, to target cells.^42,43^ Can carry misfolded proteins, namely Aβ or α-synuclein, along axonal surfaces across the brain, propagating synaptic dysfunction and injury.^44–46^ Roles in cellular communication under conditions of inflammation and injury
Apoptotic bodies	Formed by the outward budding of larger plasma membrane fractions as part of cellular decomposition in apoptotic cell death^20^	Typically larger than 500 nm. Of note, size exclusion does not entirely discriminate apoptotic bodies from exosomes.^48^ Thus, apoptotic cells may also release EVs in the exosome size	Apoptotic cell constituents including DAMPs. Typically phosphatidylserine-decorated on outer membrane surface, which predisposes them for clearing by phagocytes.^47^ Roles in cellular communication under conditions of inflammation and death

In addition to exosomes, nuclear EVs are generated by membrane budding at the inner nuclear membrane (Table 1). They are passaged through the cytosol and released into the extracellular space.^34,35^ Based on their globotriaosylceramide content, nuclear EVs can be captured by shiga toxin b subunit.^32^ Nuclear EVs are rich in pre-miRNAs. Pre-miRNAs need to be processed to miRNAs to exert biological functions.

At the endoplasmic reticulum (ER), EVs are also formed at specific membrane contact sites (Table 1). These EVs are rich in RNAs including miRNAs and mRNAs.^36^ Via direct ER-endosomal or ER-lysosomal/autophagosomal contacts, newly-formed proteins are transferred to endosomes or lysosomes/autophagosomes,^37^ via which they are further processed or released.

Under conditions of inflammation, structurally and functionally intact free and EV-encapsulated mitochondria and mitochondria fractions can be released by different cells (Table 1), including neural stem/precursor cells.^26^ These structures can restore mitochondrial function and cell metabolism of inflammatory cells, as previously shown at the example of neurons and macrophages.^26,38,39^ Structurally and functionally damaged mitochondria and mitochondria fractions can be taken up by surrounding cells for cellular degradation.^40,41^

EVs formed within cells need to be distinguished from microvesicles, which are generated by outward budding of the plasma membrane into the extracellular space (Table 1). Microvesicles typically have a diameter of 100–1000 nm.^20,22^ They possess important roles in intercellular communication, particularly under conditions of inflammation and injury. Under inflammatory conditions, microvesicles can traffic DAMPs, including interleukin (IL)-1α, IL1β or regulated and normal T cell expressed and secreted protein (RANTES), to adjacent cells, which induces cellular dysfunction and injury.^42,43^ Under neurodegenerative conditions, microglia-derived microvesicles can carry misfolded proteins, namely Aβ or α-synuclein, along axonal surfaces, propagating synaptic dysfunction across the brain.^44–46^

Apoptotic bodies with a size typically larger than 500 nm^20^ are released as part of a cellular decomposition process (Table 1). Apoptotic bodies are typically phosphatidylserine-decorated on their outer membrane surface, which predisposes them for clearing by phagocytes.^47^ Importantly, apoptotic bodies and microvesicles exhibit a broad size distribution. Thus, EV size does not entirely discriminate apoptotic bodies, microvesicles and exosomes (Table 1). Indeed, apoptotic cells may release apoptotic bodies in the exosome size. In HeLa cells, apoptotic bodies with a size clearly below 100 nm were released in staurosporine-induced apoptosis, which conferred proinflammatory signals to macrophages.^48^

The different origins of EVs imply that they carry diverse cargos and signals. Indeed, late endosomes are strongly capable to preselect their protein stocks.^20^ Despite this fact, considerable overlaps exist between protein cargos released by different EV categories.^49^ As a consequence, there are no markers available that strictly distinguish individual EV categories. In experimental settings, EV preparations furthermore represent mixtures of EVs of various cellular origins. In view of this complexity, requirements for the characterization of EVs were defined in the Minimal Information for Studies on Extracellular Vesicles guidelines.^50^ An important aspect of EVs is their size distribution. Throughout this paper, EVs within the exosome size (60–150 nm) have been referred to as small EVs. Besides this, surface marker patterns increasingly deserve attention. Of note, biological activities of EVs critically depend on cell culturing and EV separation strategies, which decisively influence EV properties in disease contexts.^50^

### Cellular interaction, uptake and signaling

The EV membrane consists of highly organized assemblies of lipids (including cholesterol and sphingolipids) and proteins, which constitute membrane microdomains. EVs abundantly contain membrane-organizing proteins named tetraspanins. Tetraspanins are a family of transmembrane proteins which contain four transmembrane domains and two extracellular loops,^51^ among which are the classical exosomal proteins CD9, CD63 and CD81 (Table 1). Although each tetraspanin exhibits different tissue and subcellular distributions, they are detected in nearly all cell types as components of plasma membranes, endosomes, and exosomes. Forming homodimers or heterodimers, tetraspanins are able to assemble to tetraspanin-enriched microdomains (TEMs) or ‘*tetraspanin webs*’. Tetraspanins arrange the spatial juxtaposition of associated transmembrane proteins and receptors on EVs.

Clustering with transmembrane integrins, selectins, cell adhesion molecules, cadherins and receptor proteins,^52^ tetraspanins provide the context-dependent tropism of EVs to injured or inflamed cells (Table 1). Membrane microdomains enrich many signaling proteins, among them several ligands and receptors, forming ligand and receptor platforms that have unique signaling properties.^53,54^ The temporally restricted interaction of membrane microdomains represents a key principle underlying intercellular communication, and the combination of surface molecules defines the membrane microdomain tropism towards selected cells.^53,55^ Thus, EVs represent mobile ligand platform carriers able to convey complex signals between cells.^18^ With shifts in EV integrin, selectin and cell adhesion molecule patterns, the EV tropism to target cells changes from the acute and chronic injury phase. Matching ligand and receptor platforms ensure that the right information is transmitted to appropriate cell types in each phase.

EVs may form transient contacts via signaling platforms and activate receptors on target cells, while retaining their vesicle integrity and shape.^56,57^ After protease-triggered resolution of cell contacts, the activated receptors may be endocytosed to transmit their signals into the cytosol.^58,59^ Endocytosis is critical for activated receptor platforms to exert their signaling responses.^60^ This transient mode of interaction is called kiss-and-run signaling.^18^

Kiss-and-run signaling has to be discriminated from cellular EV uptake via large-scale plasma membrane fusion or endosomal endocytosis, which may both result in cargo transfer.^61^ Plasma membrane fusion is impeded by the negative surface charges of EVs and target cells.^62^ The negative surface charges require that a positively charged extracellular matrix (ECM) corona enables the EV contact with target cells. The negative membrane charges explain the crucial role of positively charged proteoglycans in the mediation of synaptic plasticity.^8^ Proteoglycans, namely aggrecan and versican, exhibit complex configuration changes in the immediate vicinity of synapses during neuronal plasticity.^63–65^ These processes likely influence the uptake of EVs by target cells, besides modifying cellular interactions.

While membrane fusion enables the passage of luminal EV cargos into the cytosol, endosomal endocytosis maintains a barrier for luminal EV cargos, which need to escape the endosomal compartment and enter the cytosol to exert their function. Luminal EV cargos are effectively taken up from endosomes into the cytosol only in the presence of endosomal escape mediators.^66^ Endosomal escape proteins have recently been identified in EVs, but so far only in select conditions.^67^ These escape proteins phylogenetically mimic endosomal escape strategies of viruses enabling the delivery of virus particles into host cells.^68^

Although having the experimental knowledge that EV contents can successfully accumulate in target cells in a variety of experimental settings, our understanding of the underlying modes of uptake is still incomplete. The efficiency of defined uptake mechanisms crucially defines, to which extent cargos on the EV surface or in the EV lumen can reach their targets on the surface or inside their interacting cells.

### Roles of EVs in the initiation and propagation of neuronal injury

Being centrally involved in the regulation of neuronal survival and plasticity, EVs can have both injury promoting and inhibitory actions. Under pathophysiological conditions, EVs can transfer pro-inflammatory DAMPs to surrounding cells, as shown in a variety of brain injury models. This transfer may even occur across the blood-brain barrier (BBB), which under physiological conditions represents an efficient barrier that impedes EV passage.^69^ Indeed, brain-derived EVs may reach the blood under inflammatory conditions. Thus, in response to intracerebral IL1β injection, mostly small astroglial EVs within the size of exosomes were shown to accumulate in the blood and to promote the transmigration of leukocytes into inflammatory brain lesions via modulation of peripheral cytokine responses through inhibition of peroxisome proliferator-activated receptor-α (PPARα).^70^

Release of mostly large IL1β+ EVs within the size of microvesicles (diameter: 426 ± 51 µm) was found by cultured astrocytes upon ATP receptor P2X7 activation.^71^ In response to P2X7 activation, acid sphingomyelinase (ASM) was rapidly exposed to the outer plasma membrane leaflet. ATP-induced shedding and IL1β release were markedly reduced by ASM inhibitors, and completely blocked in astrocytic cultures obtained from ASM^−/−^ mice.^71^ Mitogen-activated protein kinase p38 was relevant for the whole process, as p38 inhibitors strongly reduced ASM activation, microvesicle shedding and IL1β release.^71^ These results provided very early demonstration that activation of ASM is necessary and sufficient for microvesicle release from glial cells, opening the way for therapeutic strategies targeting pro-inflammatory EVs. Pro-inflammatory EVs of different size likely play a role in the pathogenesis of several brain pathologies.

Under conditions of ischemia, macrophage-derived EVs of small and large size were shown to transfer the DAMPs IL1α, IL1β and RANTES to peri-infarct cells, inducing cellular dysfunction and injury.^42^ In the multiple sclerosis-like model of lysolecithin-induced axonal demyelination, the local injection of microglia-derived IL1α, IL1β and tumor necrosis factor-α (TNFα) containing small EVs prevented the remyelination of corpus callosum axons.^43^ In contrast, small EVs obtained from microglia, which were co-cultured with immunosuppressive MSCs, promoted oligodendrocyte precursor cell recruitment and myelin regeneration.

Under conditions of neurodegenerative diseases, EVs can contribute to the propagation of misfolded proteins. Small EVs with the size and protein characteristics of exosomes obtained from brain tissue of Alzheimer patients exhibited elevated levels of Aβ oligomers^72^ and hyperphosphorylated tau protein,^73^ which were shown to act as vehicles for the neuron-to-neuron transfer of Aβ oligomers and Tau protein *in vitro* and *in vivo*, respectively. Microglia-derived EVs were found to carry Aβ anterogradely along axonal surfaces.^44^ Thereby, long-term potentiation dysfunction was propagated from the entorhinal cortex to the hitherto unaffected dentate gyrus.^44^

Obtained from Parkinson’s patients, small microglia-derived EVs containing α-synuclein were shown to transport α-synuclein aggregates along axons from the striatum to the substantia nigra in mice.^45,46^ EV α-synuclein internalization was initiated by α-synuclein binding to toll-like receptor-2 (TLR2) of microglia.^46^ Depleting microglia suppressed the transmission of α-synuclein after stereotaxic injection of pre-formed α-synuclein fibrils.^45^ Thus, EVs may act as seeds of protein aggregation in remote brain areas that are so far healthy. The mechanisms of axonal EV transport are currently examined.^44^ By propagating protein folding pathologies, EVs contribute to neurodegenerative disease development.

### Roles of EVs in promoting neuronal survival and plasticity

#### EVs as mediators of neuronal plasticity

Upon brain injury, neurons, their axons, dendrites and synapses undergo profound structural and functional remodeling.^1^ Following an initial phase of retraction, axons and dendrites in the vicinity and at distance to brain lesions sprout, enabling functional neuronal network rewiring via the formation and stabilization of synapses.^74,75^ Neuronal plasticity is facilitated by axonal remyelination,^76,77^ metabolic support by astrocytes^9,78^ and trophic support by cerebral vascular cells.^79,80^ EVs decisively control cellular interactions, setting the stage for neuronal survival and plasticity in the sub-acute and post-acute injury phase.^18,81,82^

The plasticity-promoting actions of small EVs within the size of exosomes have recently been demonstrated in the perilesional cortex of rhesus monkeys exposed to motor cortical cold injury, in which intravenously delivered MSC-EVs increased dendritic branching, synaptic spine density and fine motor-coordination.^83,84^ In *in vivo* studies, immunomodulatory actions are crucially involved in the neuronal survival and plasticity-promoting effects of exogenously administered MSC-EVs. Immunomodulatory actions involve brain-invading leukocytes, namely neutrophils and monocytes/macrophages,^85–87^ and microglial cells.^84,88^ Besides, a number of genuine brain-specific actions of EVs have been discovered recently, which crucially regulate neuronal plasticity. These functions paradigmatically emphasize the importance of EVs for coordinated intercellular communication.

EV signaling vividly occurs in brain microenvironments exhibiting intense intercellular communication. Preferential sites of communication are interfaces between pre- and postsynaptic neurons at synapses,^89,90^ between astrocytes and neurons at plasma membranes or tripartite synapses,^91–94^ between oligodendrocytes and neurons at axons,^95,96^ between microglial cells/macrophages and neurons at plasma membranes or synapses,^84,88,97^ and between cerebral endothelial cells and neurons.^98,99^ At each of these interfaces, EVs safeguard neuronal integrity and function under physiological conditions and orchestrate neuronal survival and plasticity following brain injury.^100^ In the following, we first define roles of neuron-derived EVs in controlling neuronal plasticity, followed by an analysis how neuronal plasticity is modulated by EVs derived from non-neuronal cells.

### Roles of neuronal EVs in regulating neuronal plasticity

**Figure 1.**
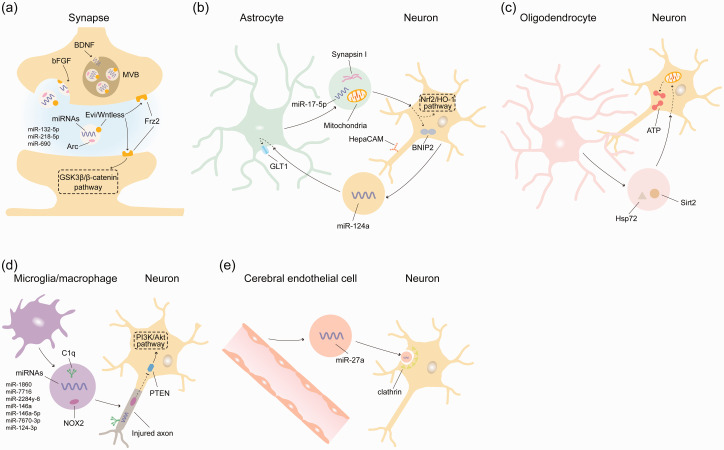
Molecular mechanisms via which endogenous extracellular vesicles (EVs) in the brain regulate neuronal survival and plasticity. Under physiological and pathophysiological conditions, various brain cells, including neurons, astrocytes, oligodendrocytes, microglia/macrophages, and cerebral endothelial cells, release small EVs carrying diverse cargo signals, such as proteins, miRNAs, and even intact mitochondria. These EVs contribute to neuronal plasticity by restoring mitochondrial energy metabolism, inhibiting excessive oxidative stress, and promoting axonal growth. (a) At the presynapse, miRNAs are packaged into small EVs by BDNF and released at the presynaptic membrane by bFGF. Neuronal EVs can coordinate synaptic growth by trafficking the Frz2 ligand Evi/Wntless to the pre- and postsynaptic membrane in a GSK3β/β-catenin-dependent manner, and by shuttling miRNAs through an Arc-dependent mechanism. In the injured brain, (b) small astrocyte-derived EVs can reduce mitochondrial oxidative stress, enhance neurite outgrowth, promote neuronal survival, and induce neuroprotection via mechanisms involving the transfer of synapsin-I and functional mitochondria to neurons, inhibition of BNIP2 by miR-17-5p, and activation of Nrf2/HO-1 signaling. The neuroprotection of astrocyte-derived EVs is mediated by HepaCAM. Conversely, neurons can regulate astrocytic GLT1 expression by delivering miR-124a-encapsulated small EVs to astrocytes, thereby promoting synaptic activity and plasticity through the control of extracellular glutamate levels. (c) Small oligodendrocyte-derived EVs can transfer Sirt2 and Hsp72 to neurons, contributing to the recovery of cellular energy metabolism, enhancement of mitochondrial integrity, and increase of intracellular ATP. (d) Microglial/macrophage-derived EVs can facilitate phagocytosis of injured axons marked by the “eat me” signal complement factor C1q. Small microglial/macrophage EVs can also transfer miRNAs and NOX2 to neuronal axons, promoting axonal regeneration via PTEN deactivation and PI3K/Akt signaling and (e) Small cerebral endothelial cell-derived EVs can be endocytosed by neurons in a clathrin-dependent manner, transferring miR-27a, which downregulates axonal inhibitory proteins through transcriptional regulation.

Synaptic contacts are sites of activity-dependent plasticity.^101^ During synaptogenesis, the plasticity of presynaptic and postsynaptic spines is mutually coordinated with each other. In this process, EV signaling was found to play a central role. Besides synaptic vesicles secreting neurotransmitters, small EVs within the size of exosomes are constantly released at presynaptic membranes in an activity-dependent way.^93^ This activity-dependent release involves syntaxin-1A (Syx1A), a protein also involved in synaptic vesicle secretion.^93^ EVs released via Syx1A were found to contain the Wingless-binding protein Evenness-interrupted (Evi)/Wntless that binds to Frizzled-2 (Frz2) at the pre- and postsynaptic membrane, inducing coordinated synaptic growth at both membranes that occurrs in a glycogen synthase kinase-3β (GSK3β)/β-catenin-dependent way^93^ (Figure 1(a)).

The EV sorting at synapses is controlled by growth factors. Brain-derived neurotrophic factor (BDNF) coordinates the sorting of miRNA stocks into neuronal EVs (Figure 1(a)). In cultured primary mouse cortical neurons, BDNF-induced TrkB activation induced the packaging of miR-132-5p, miR-218-5p, and miR-690 into EVs, which were released into the extracellular space in a neutral sphingomyelinase and ceramide-dependent way.^90^ When added to primary mouse hippocampal neurons, BDNF-induced EVs increased the formation of excitatory synapses by elevating developmental and synaptogenesis-related genes (including *Sema4a*, *Sema6c*, *Sema7a*, *Wnt7a/b*, and *NeuroD2*).^90^ BDNF-induced EVs amplified synaptic vesicle clustering, increased synaptic transmission and synchronous neuronal activity.^90^ In primary rat cortical neurons, neuron-derived EVs also increased neuronal spine density and promoted the phosphorylation of Akt and ribosomal protein S6 (RPS6).^102^ This effect was also BDNF-and TrkB-dependent. Neuron-derived EVs did not impair neuronal network activity. EVs successfully increased spine density also in neurons challenged by B27 supplement nutrient starvation.^102^

Basic fibroblast growth factor (bFGF) is a growth factor that has been known to regulate synaptic plasticity for several years.^103^ More recent studies showed that bFGF controls EV release from cultured neurons (Figure 1(a)). Rat hippocampal neurons only exhibit modest EV release from late endosomes/multivesicular bodies (MVBs) during spontaneous electrophysiological activity.^89^ Electrical stimulation increased neuronal EV release. This activity-dependent release was significantly elevated by bFGF delivery.^89^ Proteome analysis showed that bFGF increased the EV abundance of vesicle-associated membrane protein-3 (VAMP3).^89^ VAMP3 knockdown in cultured neurons reversed the effect of bFGF on EV release, indicating a crucial role of VAMP3 in activity-dependent EV secretion.

The loading of miRNA and mRNA into EVs involves specialized packaging machineries. The cytoskeleton-associated protein Arc is a master regulator of synaptic plasticity in mammals and is required for protein synthesis-dependent forms of long-term potentiation (LTP) and depression (LTD).^104,105^ Arc is capable of self-assembling into virus-like structures with a size of 20–60 nm that encapsulate RNA.^67^ Endogenous Arc protein is released in EVs from mouse hippocampal neurons that mediate the transfer of mRNA into new target cells, where it can undergo activity-dependent translation^67^ (Figure 1(a)). Purified Arc capsids are endocytosed and able to transfer mRNA into the cytoplasm of neurons.^67^ Structurally, Arc exhibits similar molecular properties to retroviral Gag retrotransposons.^106^ This suggests that Arc may have been repurposed phylogenetically to mediate intercellular communication in the brain.^67^ Arc might provide an endosomal packaging and escape mechanism, via which miRNA and mRNA can be exchanged between cells.

For maintaining activity-dependent plasticity, synaptic spines require stocks of release-competent endosomes containing EVs with adequate signaling cargos. The formation of such stocks relies on the functionality of the endocytic machinery, which comprises proteins such as nervous wreck (Nwk), shibire/dynamin and AP2 adaptor complex.^107^ The deficiency of these proteins locally depleted EV stocks from presynaptic terminals, as shown in *Drosophila*. Thus, *Nwk* mutants exhibited synaptic plasticity defects phenocopying those associated with deficiency of synaptotagmin-4 (SYT4), a known EV cargo.^107^ Mechanistically, Nwk assisted in the loading of cargos into EVs. Activity-dependent synaptic EV signaling has not been modulated therapeutically in the injured brain. Synaptic EV responses might represent a promising target for enhancing use-dependent plasticity, e.g., under conditions of neurorehabilitation.

At tripartite synapses, extracellular glutamate levels inside the synapse are controlled by astrocytic processes, which express glutamate transporters, namely glutamate transporter-1 (GLT1). By releasing small EVs within the size of exosomes, neurons are capable of controlling astrocytic GLT1 protein levels, thereby modulating synaptic activity and plasticity (Figure 1(b)). Thus, EVs released from primary mouse neurons were found to increase GLT1 protein levels on astrocytes via mechanisms involving miR-124a transfer.^94^ Intrastriatal injection of antisense RNA against miR-124a reduced GLT1 protein expression and glutamate uptake in the adult mouse striatum, yet without reducing *Glt1* mRNA levels.^94^ The miR-124a-mediated regulation of GLT1 appeared to be indirect and not mediated by its suppression of the putative GLT1 inhibitory ligand ephrinA3. In spinal cords of endstage SOD1 G93A mice, an amyotrophic lateral sclerosis model, miR-124a was reduced.^94^ The stereotactic injection of miR-124a prevented the pathologic loss of GLT1 protein in spinal cord astrocytes of SOD1 G93A mice.^94^

When considering the signaling properties of EVs of primary neurons obtained during early neuronal development, which are widely used *for in vitro* studies, it needs to be considered that their EV cargos substantially differ from adult neurons *in vivo.*^108^ Such differences specifically relate to small non-coding RNAs, including miRNAs, which are spatially highly regulated, as shown in a comprehensive study comparing miRNA contents *in vitro* and *in vivo* at the example of cortical neuronal axons and their EVs.^108^

### Roles of non-neuronal EVs in regulating neuronal plasticity

#### Roles of astroglial EVs

Besides regulating neurotransmitter levels, astrocytes control neuronal energy metabolism by lactate shuttling.^9^ In addition, astrocytes have trophic activities, enabling neuronal survival and plasticity via EVs^9^ (Figure 1(b)). The oligomannose-mimicking peptide synapsin-I is a neurite growth stimulant released by mouse astrocytes through small EVs within the size of exosomes. When transferred to neurons, astroglial synapsin-I was shown to increase neurite outgrowth and to promote neuronal survival after hydrogen peroxide exposure or oxygen-glucose deprivation.^91^ Co-cultures of wildtype or synapsin-I-deficient glial cells with wildtype neurons revealed that synapsin-I enhanced neurite outgrowth when expressed on glial cells. Synapsin-I-induced neurite outgrowth was dependent on oligomannose residues on synapsin-I and on neural cell adhesion molecule (NCAM) expression at the neuronal cell surface.^91^

Thus, the promotion of axonal plasticity by astroglial EVs involves surface contact mechanisms (Figure 1(b)). Following motor cortical injection, EVs obtained from astroglial cells were found to spread over long distances along the corticospinal tract in adult and early postnatal developing mice. In early postnatal mice, small astroglial EVs induced axonal growth and increased growth cone size of cortical pyramidal neurons.^109^ The growth-stimulating effects of astroglial EVs were mediated by the surface expression of hepatocyte cell adhesion molecule (HepaCAM, also known as glial cell adhesion molecule), an adhesion molecule containing immunoglobulin (Ig)-like extracellular domains that interacts with ECM components.^109^ Interestingly, apolipoprotein-E (ApoE), which is also released by astroglia, abolished the stimulating effects of astroglial EVs on axon outgrowth.^109^ These studies suggest that astroglial signals can have both stimulating and inhibitory effects on axonal plasticity, and that EV-bound growth stimulants antagonize with lipoproteins to exert these actions.

#### Roles of oligodendroglial EVs

Neurons possess long axons that require the provision of energy-rich substrates and trophic factors that enable their long-term stability and function.^110^ The long-term integrity of axons depends on intrinsic axonal transport mechanisms and extrinsic support mechanisms by adjacent oligodendroglia (Figure 1(c)). Oligodendroglia were found to release small EVs with the size and protein characteristics of exosomes, which when delivered to primary mouse hippocampal neurons cultured under regular conditions or oxidative stress conditions increased the anterograde transport of BDNF-mCherry-carrying axonal vesicles.^95^ When applied to hippocampal neurons exposed to nutrient (B27 supplement) starvation, in which axonal transport was significantly reduced, oligodendroglial EVs almost completely restored the anterograde and retrograde transport of axonal vesicles.^95^ EV release was reduced in cultured oligodendrocytes obtained from proteolipid protein (*Plp*)^−/−^ and 2′,3′-cyclic-nucleotide-3′-phosphodiesterase (*Cnp*)*^−/−^* mice exhibiting progressive corticospinal degeneration.^95^ Anterograde and retrograde axonal transport were compromized in *Plp*^−/−^ and *Cnp^−/−^* mice.^95^ Mutant EVs lacked the ability to restore anterograde and retrograde axonal transport in nutrient-deprived primary mouse cortical neurons.^95^ These findings were interpreted that glia-to-neuron EV transfer promotes the long-term maintenance of neurons by facilitating axonal transport, providing a novel mechanistic link between myelin diseases and secondary loss of axonal integrity.

#### Roles of microglial and macrophage EVs

Microglia are brain-intrinsic tissue resident macrophages, which in addition to blood-derived macrophages control neuronal plasticity.^64,111^ An important mechanism, via which microglia/macrophages promote plasticity, is the proteolytic digestion of ECM that restricts synaptogenesis.^64^ Microglia/macrophages are also capable of phagocytosing synapses or neurons destined for removal.^111^ These synapses are tagged with the eat me signal complement factor C1q, which activates phagocytic programs in microglia/macrophages.^111^

Microglial EVs are involved in neuronal C1q tagging, as shown in rats with fetal alcohol exposure, in which EVs with β-endorphin neuronal killing activity were isolated from hypothalamic brain tissue, which had the size and appearance of exosomes.^112^ EV proteome analysis followed by protein assays identified a large number of proteins, including various complements, which were elevated on microglial EVs by ethanol exposure.^112^ Ethanol exposure increased the deposition of C1q on β-endorphin neurons *in vitro* and *in vivo*. Recombinant C1q protein increased, while C1q blockers reduced ethanol-induced C3a/b, C4 and membrane attack complex/C5b9 formation, ROS production, and ultimately β-endorphin neuronal death.^112^ These data suggest that microglial EVs are capable of C1q tagging of neurons or neuronal membranes, predisposing the se neurons for the removal by activated microglia/macrophages (Figure 1(d)). The removal of damaged synapses sets the stage for new synapse growth.

During neuronal sprouting, microglial/macrophage EVs regulate neuronal plasticity processes by delivering specific cargos to neurons. The regulation of plasticity requires the communication of axons with their somas and nuclei. In a mouse model of sciatic nerve injury, macrophages recruited to neuronal lesions by reactive oxygen species were shown to release small EVs within the size of exosomes containing functional NADPH oxidase-2 (NOX2), a producer of NO radical.^97^ These NOX2+ EVs were taken up by injured axons via endocytosis and were retrogradely transported to the soma in axonal endosomes.^97^ The retrograde NOX2 transport involved an importin-β1-dynein-dependent mechanism. In the soma, NOX2 oxidized phosphatase and tensin homolog (PTEN), leading to its inactivation, as shown in dorsal root ganglion cell cultures.^97^ Inactive PTEN stimulated phosphatidylinositol-3 kinase (PI3K)/Akt signaling, resulting in axonal growth (Figure 1(d)).

In addition to enzymes and proteins, the EV-mediated communication between axons and their soma involves miRNAs, which are spatially highly regulated along axons^108^ and can either promote or inhibit synaptic plasticity, depending on the nature and differentiation of the EV-secreting cells. Using advanced isolation techniques combining ultracentrifugation with Optiprep density gradient separation, RNAse-A treatment for destroying surface-bound RNA and size exclusion separation, sets of miRNAs were assessed in non-stimulated small EVs obtained from leech microglia, which increased neurite outgrowth of rat primary neurons.^113^ miR-146a, miR-1860 miR-2284y-6 and miR-7718, were found to be enriched in neurite growth-promoting microglial EVs^113^ (Figure 1(d)).

In another study using stimulated M1-like pro-inflammatory rat microglia, miR-146a-5p was increased in large EVs within the size of microvesicles.^114^ miR-146a-5p was taken up by primary hippocampal neurons (Figure 1(d)) to decrease the expression of presynaptic synaptotagmin1 (Syt1) and postsynaptic neuroligin1 (Nlg1), an adhesion protein with a role in dendritic spine formation and synaptic stability.^114^ Microglia-to-neuron miR-146a-5p transfer and Syt1 and Nlg1 downregulation did not occur when EV-neuron contact was inhibited by blocking vesicular surface phosphatidylserine residues with annexin-V, when EVs were depleted of miR-146a-5p, when EVs stored inactive miR-146a-5p, or when EVs were produced by cells transfected with miR-146a-5p antagomir.^114^ Morphological analysis revealed that prolonged exposure to miR-146a-5p-enriched EVs decreased dendritic spine density of hippocampal neurons *in vivo* and *in vitro*. The opposite roles of miR-146a in the two studies require further examination. Differences in species, EV size and category might explain the different roles of miR-146a in the two settings. The latter study investigated microvesicles with strong pro-inflammatory (surface phosphatidylserine+) properties.

#### Roles of cerebral microvascular endothelial EVs

Cerebral microvascular endothelial cells transport oxygen and nutrients to the brain parenchyma and control brain microenvironments.^115,116^ At the same time, they provide trophic support to promote brain plasticity and tissue remodeling.^117,118^ EVs released by endothelial cells contribute to this process. In primary rat cortical neurons kept in a multicompartmental cell culture system, small EVs within the size of exosomes, which were obtained from ischemic cerebral endothelial cells of rats sacrificed 7 days after transient MCAO, more efficiently stimulated axonal growth than cerebral endothelial EVs obtained from non-ischemic rats.^80^ EVs were internalized by axons in order to retrogradely transfer miRNAs to the nucleus, as shown by GFP labelling followed by immunogold detection.^80^ The transferred miRNAs downregulated axonal growth inhibitors in the soma via gene expression repression.^80^ Blockade of axonal transport suppressed cerebral endothelial EV-associated miRNA actions on axonal growth inhibitor expression in the soma.

In mice exposed to permanent MCAO, a specific role of miR-27a in this cellular signaling process was demonstrated^99^ (Figure 1(e)). Small EVs within the size of exosomes obtained from cerebral endothelial cells that were lentivirally transfected with miR-27a more efficiently promoted post-ischemic corticospinal plasticity and improved neurological outcome compared to EVs from cerebral endothelial cells transfected with scramble miRNA.^99^ Ultrastructural analysis revealed that systemically administered miR-27a-enriched small EVs preferentially accumulated in the pre-synaptic active zone. Quantitative reverse transcription-polymerase chain reaction and Western blot analysis showed elevated miR-27a expression in the peri-infarct tissue, accompanied by reduced expression of the axonal inhibitory proteins semaphorin 6 A and Ras homolog family member A.^99^ Blockage of clathrin-dependent endocytosis reduced the internalization of miR-27a-enriched small EVs.

### Mitochondrial dysfunction and associated pathological events as EV targets

**Figure 2.**
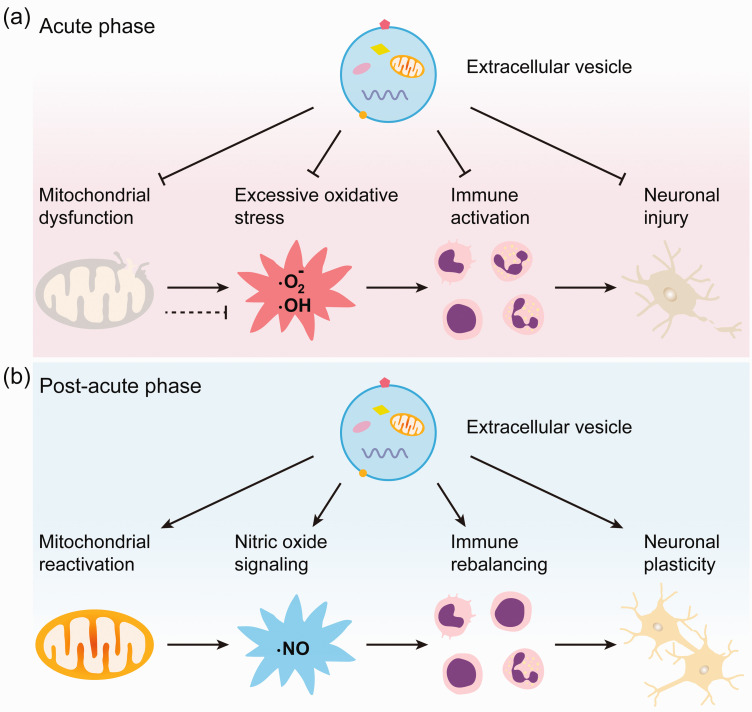
Small EVs orchestrate neuronal survival and plasticity across different stages of injury. (a) During the acute phase of injury, small EVs within the size of exosomes can restore disrupted mitochondrial energy metabolism and integrity, suppress mitochondrial dysfunction-induced oxidative stress, and modulate the overactive immune response, thereby promoting neuronal survival and (b) in the post-acute phase, small EVs can facilitate mitochondrial reactivation, enhance neuroregenerative nitric oxide signaling, and regulate immune responses to maintain a balance between overactivation and suppression, thereby supporting neuronal plasticity.

Neurons rapidly reduce mitochondrial energy metabolism in response to injury as a self-protection mechanism.^119^ Mitochondrial inhibition occurs by the post-translational regulation of signals that broadly impact cell metabolism. These signals include hypoxia-inducible factor 1α (HIF1α), peroxisome proliferator-activated receptor γ coactivator α (PGC-1α), c-Myc and sirtuin-1 (Sirt1).^119^ Dysregulated mitochondrial function is a major hazard to injured neurons (Figure 2(a)). Uncoupling of mitochondrial electron transport gives rise to excessive oxidative stress,^120,121^ which increases mitochondrial calcium accumulation, exacerbates mitochondrial damage and compromises neuronal survival and plasticity.^122–124^ Once a critical threshold of mitochondrial damage is exceeded, mitochondrial permeability transition pores (MPTP) are formed, which release ROS and cytochrome-c into the cytoplasm inducing apoptotic death via caspase-9 and -3 activation.^125^

Of note, oxidative stress is not always detrimental to the brain. Under physiological conditions, oxidative stress supports neuronal plasticity processes (Figure 2(b)). Thus, NO radicals generated in response to synaptic activity are capable to promote the structural and functional plasticity of neurons.^126^ NOX2, which catalyzes NO radical formation and can be transferred by small macrophage-derived EVs within the size of exosomes to neurons, thereby stimulating axonal growth, as demonstrated in a mouse sciatic nerve injury model.^97^

In principle, EVs are able to regulate cell energy metabolism in two ways, i.e., by modulating mitochondrial integrity and function and by attenuating oxidative stress, inflammation and death processes (apoptosis, pyroptosis, ferroptosis) associated with mitochondrial injury (Figure 2). Both aspects are evaluated in the following.

### Mitochondrial dysfunction and apoptosis as EV targets

In the acute phase of ischemic injury, the balance of anti-apoptotic Bcl-2 family proteins (e.g., Bcl-2, Bcl-X_L_) and pro-apoptotic Bcl-2 family proteins (e.g., Bax) in mitochondria shifts towards pro-apoptotic Bcl-2 proteins,^125^ which sets the stage for MPTP formation and caspase-3-dependent apoptosis, once a critical degree of mitochondrial damage is reached.^125^ In hippocampal neurons exposed to OGD and rats exposed to MCAO, the delivery of MSC-EVs induced neuroprotection by mechanisms involving the restoration of Bcl-2/Bax balance, which inhibited caspase-3-dependent apoptosis.^127,128^ In one of these studies,^127^ this effect was mediated by elevating miR-93 levels that suppressed the expression of histone deacetylase-4 (HDAC4), which is highly abundant and active in ischemic brain tissue. In another study,^128^ miRNA sequencing and functional enrichment analysis identified miR-877-3p as a key component of neuroprotection by small dental pulp-derived MSC-EVs. In a dual-luciferase assay, miR-877-3p was found to interact with Bcl-2-associated transcription factor (Bclaf1) to regulate the expression of Bcl-2 family proteins.^128^ The miR-877-3p inhibitor or Bclaf1 overexpression reversed the neuroprotective effects of dental pulp-derived MSC-EVs.^128^ In neonatal rats exposed to hypoxic-ischemic brain injury, astrocyte-derived EVs induced neuroprotection and reduced oxidative stress by restoring Bcl-2/Bax balance.^129^ This regulation involved miR-17-5p binding to Bcl-2-interacting protein-2 (BNIP2), which downregulated BNIP2 expression in ischemic cells^129^ (Figure 1(b)).

The recovery of energy metabolism following injury depends on the ability of the NAD-dependent deacetylase Sirt2, which is localized in mitochondria and controls energy metabolism, oxidative stress and DNA repair,^130^ and of the chaperone heat shock protein-72 (Hsp72), which restores misfolded proteins,^131^ to reestablish mitochondrial integrity (Figure 1(c)). In the central nervous system (CNS), axons are particularly vulnerable sites predisposed to metabolic disturbances. In the spinal cords of *Sirt2^−/−^* mice, small EVs within the size of exosomes obtained from wildtype oligodendrocytes were found to transfer Sirt2 to neurons, where they induced mitochondrial adenine nucleotide translocase-1/2 (Ant1/2) deacetylation, increased intracellular ATP level and enhanced mitochondrial integrity.^96^ EVs obtained from *Sirt2^−/−^* oligodendrocytes did not exert these actions in *Sirt2^−/−^* mouse spinal cords.^96^ Deficient oligodendrocytic metabolic support was also made responsible for the progressive axonal degeneration in *Plp*^−/−^ and *Cnp*^−^*^/^*^−^ mice.^95^ Small EVs of *Plp*^−/−^ and *Cnp*^−^*^/^*^−^ oligodendrocytes revealed reduced Sirt2 and Hsp72 levels compared with wildtype oligodendrocyte EVs.^95^ When transferred to primary cortical neurons exposed to nutrient deprivation, EVs of *Plp*^−/−^ and *Cnp*^−^*^/^*^−^ oligodendrocytes were unable to rescue neuronal metabolic activity evaluated by the 3-(4,5-dimethylthiazol-2-yl)-5-(3-carboxymethoxyphenyl)-2-(4-sulfophenyl)-2H-tetrazolium (MTT) assay.^95^ In contrast, EVs obtained from wildtype oligodendrocyte EVs restored metabolic activity, enabling neuronal survival.^95^

In view of their structural damage, injured neurons lack functional proteins and enzymes required for maintaining energy metabolism. EVs are highly enriched in glucose transporters and glycolytic enzymes, as demonstrated in cardiomyocytes exposed to glucose deprivation.^23^ These EVs increased glucose uptake, glycolysis, and pyruvate production in recipient endothelial cells.^23^ EVs may also transfer enzymes involved in ATP turnover (e.g., adenylate kinase, ATPase, 5′-nucleotidase), which contribute to ATP formation when supplied with their substrates, as demonstrated for EVs produced by prostate cells (exosome-like prostasomes).^24^ In cancer, up to one quarter of proteins enriched in cancer-derived large EVs (i.e., oncosomes) are enzymes involved in glucose, glutamine, and amino acid metabolism.^132^ These proteins are highly relevant for cancer progression.^133^ Via EV-bound amino acids and tricarboxylic cycle intermediates, tumors induce a metabolic switch from oxidative phosphorylation to glycolysis.^134^ The resulting lactate is utilized by cancer cells to promote tumor growth. Although significantly less is currently known for brain cells compared to cancer, the responses of the ischemic brain strikingly resemble those of cancer cells. Post-ischemia, shifts in oxidative phosphorylation/glycolysis balance control neuronal survival and synaptic plasticity through astrocytes.^9^

Besides carrying miRNAs and proteins, EVs can transfer mitochondrial membrane fragments and even entire mitochondria to neighboring cells^38,39^ (Figure 1(b)). Thereby, EVs may help unload injured mitochondria from stressed cells in a process termed transmitophagy. This mechanism was, for example, demonstrated in retinal ganglion cell axons releasing acidified mitochondria associated with lysosomes, which were taken up by adjacent astrocytes for degradation.^40^ Lysosomal uptake protects the cells against inflammatory responses elicited by oxidized mitochondrial proteins.^41^ The Parkinson’s disease-associated protein parkin recognizes damaged mitochondrial proteins and membrane fractions and directs them to the lysosomes.^41^ Less severely injured mitochondria may be reutilized by recipient cells: Thus, depolarized mitochondria released from MSCs via EVs were engulfed and restored by macrophages and regained bioenergetic function.^38^ Upon ischemia, astrocytes can release functionally intact mitochondria by a calcium-dependent mechanism involving CD38/cyclic ADP-ribose signaling, which are transferred to adjacent neurons.^39^ When administered to MCAO mice, the mitochondrial transfer increased cellular ATP levels, neuronal survival, and dendritic growth.^39^ CD38 knockdown reduced cellular mitochondrial transfer and worsened neurological outcome.^39^ Endothelial precursor cells similarly can release viable mitochondria, which are taken up by brain endothelial cells, increasing intracellular ATP levels, preserving microvascular integrity and promoting angiogenesis.^135^ Mitochondrial transfer is not the major focus of this paper. For further information, readers are referred to the contribution of Peruzzotti-Jametti and Pluchino in this special issue.

### Oxidative stress and inflammation as EV targets

Oxidative stress was found to trigger delayed neuronal degeneration, brain atrophy and cognitive impairment in rat traumatic brain injury models.^136^ In these models, small astroglial EVs attenuated mitochondrial oxidative stress, neuronal loss and brain atrophy by activating nuclear factor erythroid-2-related factor-2 (Nrf2)/heme oxygenase-1 (HO-1) signaling and increasing antioxidant superoxide dismutase (SOD) and catalase activity^136^ (Figure 1(b)). The neuroprotective effects of astroglial EVs were abrogated in brain-specific *Nrf2*^−^*^/^*^−^ mice. In a mouse transient MCAO model, small bone marrow-derived MSC-EVs transcriptionally increased the expression of acyl-CoA synthetase long-chain family member-4 (Lin28a) in ischemic brain tissue in an Nrf2-dependent way.^137^ This effect was mimicked by Lin28a overexpression. In a model of H_2_O_2_-induced oxidative stress *in vitro* and methotrexate-induced neuronal damage in rats *in vivo*, small adipose MSC-EVs reduced MTX-induced hippocampal neuronal damage and reduced ROS production via Nrf2 activation, decreasing pro-inflammatory IL6, TNFα and IFNγ levels.^138^ In rats exposed to bilateral common carotid artery occlusion, a model of vascular dementia, small umbilical cord-derived MSC-EVs decreased oxidative stress, microglial M1 polarization, pro-inflammatory cytokine levels and histological brain damage in an phosphatidyl-inositol-3-kinase/Akt/Nrf2-dependent way.^139^

Microglia are sensors of cell injury and neuroinflammation, which may stabilize or destabilize neuronal integrity and plasticity, depending on their cellular polarization state. In murine BV2 and human primary microglia, HIV-1 Tat protein induced the expression of NLRP3 and IL1β, which were packaged into small EVs and released into the extracellular space.^140^ In primary rat neurons, Tat-stimulated NLRP+ and IL1β+ EVs downregulated the synaptic proteins postsynaptic density protein-95 (Psd95), synaptophysin and vesicular glutamate transporter-1 (vGLUT1), induced a loss of dendritic spines and functionally impaired miniature excitatory postsynaptic currents (mEPSCs).^140^ To assess the role of NLRP3 in this process, neurons were exposed to EVs from Tat-exposed NLRP3-silenced microglia, which exerted a protective effect on neuronal synaptic proteins, spine density and mEPSCs.^140^ Protective effects on dendritic spine integrity were also observed for EVs obtained from anti-inflammatory M2-like microglia polarized by 1070 nm near-infrared light exposure, which was known to alleviate β-amyloid burden and improve cognitive function in 5xFAD mice.^141^ M2 microglia were found to release small EVs containing miR-7670-3p, which reduced activating transcription factor-6 (Atf6) expression to attenuate ER stress, decrease brain inflammatory responses and preserve dendritic spine integrity of cortical and hippocampal neurons.^141^ In a model of glutamate-induced neuronal injury of mouse hippocampal HT22 cells, small EVs obtained from M2-polarized murine BV2 microglia were found to reduce apoptosis, restore mitochondrial membrane potential, reduce ROS accumulation and increase anti-oxidant capacity presumably via miR-124-3p^142^ (Figure 1(d)). The protective effects of M2-microglial EVs on neurons were replicated by miR-124-3p mimic.^142^ In mice exposed to MCAO, small EVs obtained from hypoxically preconditioned M2-like microglia decreased peri-infarct brain edema, pro-inflammatory cytokine responses and astrogliosis, while AQP4 polarization on astrocytic endfeet, CSF flow and neurological recovery were increased.^143^ Furthermore, pre-conditioned M2-like microglial EVs induced a shift from M1 to M2 microglia polarization.^143^

Attracted by DAMPs released by injured neurons, peripheral blood leukocytes, namely polymorphonuclear neutrophils, monocytes, T and B lymphocytes, invade the injured brain and exacerbate neuronal damage^144–146^ (Figure 2(a)). Since EVs are rapidly taken up within minutes after systemic delivery by leukocytes (specifically by monocytes, neutrophils and B lymphocytes),^147^ leukocytes might represent attractive targets for EVs in clinical settings.^18^ In MCAO mice, the effects of small bone marrow-derived MSC-EVs on neurological deficits and ischemic injury were closely associated with anti-inflammatory actions of EVs, namely the prevention of neutrophil, monocyte/macrophage and lymphocyte brain entry.^85,86^ Neutrophil depletion by delivery of an antibody against the neutrophil-specific antigen Ly6G mimicked the effects of intravenously administered MSC-EVs on neurological deficits, ischemic injury, brain monocyte/macrophage and lymphocyte infiltrates.^86^ In neutrophil-depleted mice, MSC-EVs did not have any additional effect on neurological deficits and ischemic injury, and brain monocyte/macrophage and lymphocyte infiltrates were not reduced by MSC-EVs.^86^ Notably, the role of neutrophils in mediating post-ischemic actions of MSC-EVs was not limited to the acute stroke phase. When administered in the post-acute stroke phase, from 24 hours to 5 days post-MCAO, small EVs obtained from hypoxic MSCs were found to promote peri-infarct angiogenesis.^87^ These angiogenic effects were abolished when the same hypoxic MSC-EVs were administered in neutrophil-depleted mice.^87^ Neutrophils are early brain invaders after MCAO, which exacerbate ischemic damage in the acute stroke phase, but may support brain tissue remodeling in the post-acute phase,^148^ which probably explains the impact of the treatment timing on EV responses.

### Ceramide and ceramide-rich membrane platforms as EV targets

In the signaling of cellular stress responses, the formation of ceramide-rich platforms on plasma membranes is a critical event. Ceramide is a sphingolipid that consists of a sphingoid base that is linked to a fatty acid.^149^ Upon radiation injury, UV light exposure or reperfusion damage, ceramide is generated within minutes through hydrolysis of sphingomyelin,^150,151^ which is mediated by ASM on the outer plasma membrane leaflet and by neutral sphingomyelinase (NSM) on the inner plasma membrane leaflet. Ceramide-rich platforms are enriched in transmembrane proteins, including death receptors, amplifying protein oligomerization and activation.^152^ CD95 clustering on ceramide-rich platforms precedes death-inducing signaling complex (DISC) formation and caspase activation on a variety of cells,^153^ which can be prevented by ASM inhibitors, which are clinically used as antidepressants.^154^ NSM and ASM critically control the budding of intraluminal vesicles in late endosomes, which are released into the extracellular space as exosomes, in a way that NSM promotes EV formation, whereas ASM prevents it.^98,155^ Importantly, both, EV release and composition, are profoundly altered by NSM and ASM inhibitors.^98,155^ NSM and ASM deactivation have therefore been identified as tools to regulate tissue EV numbers and cargos.

In mice exposed to MCAO, the antidepressants amitriptyline and fluoxetine increased post-ischemic angiogenesis assessed by microvascular length and branching point density in the reperfused brain tissue within 14-28 days.^98^ This effect was ASM-dependent, since amitriptyline did not increase angiogenesis in ASM^−/−^ mice.^98^ The increased angiogenesis induced by both antidepressants was accompanied by increased neuronal survival, increased BBB integrity, reduced brain infiltration of CD45^+^ leukocytes and, in case of fluoxetine, enhanced neurological recovery.^98^ Studies on human cerebral microvascular endothelial cells (hCMEC/D3) showed that amitriptyline stimulated the release of small EVs from endothelial cells within the size of exosomes that were able to increase angiogenesis in hCMEC/D3 cells very similar to amitriptyline.^98^ These studies indicated that small endothelial EVs mediated the pro-angiogenic and restorative effects of ASM inhibitors.

Evidence confirming the relevance of ASM-related EV signaling comes from studies in 5xFAD Alzheimer’s mice, in which ASM-dependent ceramide formation triggered pro-inflammatory cytokine (TNF-α, IL-1α) secretion by microglia, which induced the release of ceramide-rich small and large EVs by reactive astrocytes, which in turn impeded the mitochondrial respiration of neurons.^156^ ASM inhibition by imipramine reduced Alzheimer’s pathology and microglial cytokine secretion.^156^ In 5xFAD mice, brain derived-EVs of mice treated with imipramine contained reduced levels of the astrocytic marker GFAP, ceramide, and Aβ and did not impair mitochondrial respiration compared to EVs derived from untreated 5xFAD mice.^156^

Ceramide formation via NSM2 plays an important role in cell senescence in humans, resulting in the accumulation of very long-chain 24:1 ceramide on serum EVs that induce senescence markers on MSCs.^157^ In middle-aged (10-15-month-old) NSM2^−/−^ mice, levels of the senescence markers C3b and p27 and the pro-inflammatory cytokines IL1β, IL6 and TNFα were reduced in the cerebral cortex compared with NSM2^+/−^ mice, concurrent with twofold decreased phosphorylation of their downstream target, signal transducer and activator of transcription-3 (Stat3).^158^ Oxidative stress induced by tertiary butyl peroxide decreased the level of glutathione, an endogenous NSM2 inhibitor, and increased ceramide levels in primary NSM2^+/−^ astrocytes, but not in NSM2^−/−^ astrocytes.^158^ RNA sequencing analyses revealed that transcripts involved in mitochondrial oxidative phosphorylation and astrocyte activation were reduced in the cortex of NSM2^−/−^ compared with NSM2^+/−^ mice, while axon guidance and synaptic plasticity transcripts were increased.^158^ The signaling responses in neurons were interpreted to be EV-dependent. The total number of EVs was decreased 4-fold in the cortex of NSM2^−/−^ compared with NSM2^+/−^ mice.^158^

### Pyroptosis and ferroptosis as EV targets

Via the activation of membrane pattern receptors, such as toll-like receptor-4 (TLR4), DAMPs are capable of activating the NFκB signaling pathway in injured microglial cells and neurons. Once a critical level of cell injury is reached, this pathway triggers pyroptosis via NLRP3 inflammasome activation resulting in caspase-1, IL1β and gasdermin-D cleavage, which induces plasma membrane pore formation and cell death.^159^ In rats exposed to permanent distal MCAO, small EVs obtained from blood plasma of rats intraperitoneally treated with the free radical scavenger melatonin reduced ischemic injury and enhanced neurological recovery by decreasing NLRP3 inflammasome activation in microglia and neurons, evidenced by decreased NLRP3, apoptosis-associated speck-like protein, activated caspase-1 and cleaved gasdermin-D levels.^160^ These effects were likely mediated in an TLR4 and NFκB dependent way.^160^ Small EVs obtained from blood plasma of rats not treated with melatonin less effectively reduced ischemic injury, neurological impairments and NLRP3 inflammasome activation in the same study.

Damaged or dying neurons destined for phagocytic removal were shown to express osteopontin, a secreted phosphoprotein known to contribute to wound repair.^161^ In mice intrastriatally injected with the DAMP ATP, osteopontin+ neurons were phagocytosed by monocytes, which subsequently revealed inflammasome activation.^162^ Activated monocytes in turn released small CD63+ osteopontin+ EVs, which induced neuron and astrocyte process elongation towards the site of injury and enhanced neurite outgrowth of cultured neurons in an osteopontin-dependent way.^162^ Osteopontin expression and monocytic pyroptosis were decreased in leucine-rich repeat kinase-2 (LRRK2) G2019S compared with wildtype mice, which exhibited decreased release of small osteopontin+ EVs and decreased neuron and astrocyte process elongation.^163^ The LRRK2 G2019S mutation is the most prevalent mutation in patients with sporadic and familial Parkinson’s disease.^164^ These results suggest that severely injured neurons can activate neuronal outgrowth programs upon exposure to small monocyte EVs, which might enable repair processes.

Under conditions of massive oxidative stress, lipid peroxidation induces the failure of glutathione-dependent antioxidant defenses, resulting in uncontrolled lipid peroxidation and ferroptotic cell death.^159^ Lipophilic antioxidants and iron chelators can prevent ferroptosis, since this form of cell death crucially depends on catalytic oxidation processes involving Fe^2+^ iron (so-called Fenton reaction).^159^ In MCAO rats, preconditioning exercise before stroke decreased infarct area, improved neurological function and attenuated ferroptosis, defined by reduced lipid peroxidation, increased glutathione peroxidase-4 (Gpx4) and elevated levels of solute carrier family 7 member 11 (Slc7A11), which imports the glutathione precursor cysteine into cells.^165^ The protective effects of preconditioning exercise were associated with reduced acyl-CoA synthetase long-chain family member 4 (Acsl4), which predefines ferroptosis sensitivity by shaping cellular lipid composition.^166^ Dual luciferase reporter assays revealed that exercise-induced small EV-bound miR-484, which was mainly derived from skeletal muscle, inhibited *Acsl4* expression.^165^ Of note, the neuroprotective effect of preconditioning exercise was suppressed by inhibiting miR-484 production in skeletal muscle.^165^

In MCAO mice, intranasally administered small adipose MSC-EVs reduced ischemic injury, neurological deficits and ferroptosis by increasing Gpx4 protein level and decreasing malondialdehyde, Acsl4 protein and cerebral Fe^2+^ levels.^167^ In mouse N2a neuroblastoma cells exposed to OGD, the inhibition of ferroptosis was mediated by EV-bound miR-760-3p, which reduced the expression of glutathione-specific gamma-glutamyl cyclotransferase (Chac1), which catalyzes the cleavage of glutathione into 5-oxo-L-proline and a Cys-Gly dipeptide.^167^ This study emphasized the role of Chac1 in post-ischemic ferroptosis.

### Overarching considerations and outlook

This review identified two main trajectories, via which EVs regulate neuronal survival and plasticity. First, endogenous EVs crucially control neuronal survival and plasticity under physiological and pathophysiological conditions (Figure 1). Predisposed sites of endogenous EV signaling in the brain are pre- and post-synaptic spines,^89,90^ interfaces of astroglia and oligodendroglia with neurons at tripartite synapses and along axons,^91–96^ interfaces of microglia and monocytes/macrophages with neurons,^84,88,97^ and interfaces of the cerebral microvasculature and brain parenchymal cells.^98,99^ At each of these interfaces, EVs safeguard neuronal survival and coordinate neuronal plasticity responses. The endogenous modes of action of EVs can be leveraged therapeutically under conditions of brain ischemia, trauma or neurodegenerative diseases to protect the injured tissue against secondary damage, promote plasticity and enhance disease outcome, as shown by a large number of studies in this review.^27,28,30,31,136^

Mitochondrial disturbances are a joint hallmark of cell injury under ischemic, traumatic and neurodegenerative conditions, which critically endanger the survival of brain tissue and result in long-term functional impairments. In the ischemic brain, disturbances of energy metabolism are directly responsible for neuronal death and brain infarct development.^168,169^ In brain trauma and neurodegenerative diseases, mitochondrial dysfunction, oxidative stress and inflammation contribute to secondary brain injury.^170,171^ Thus, secondly, the promotion of neuronal survival and plasticity by EVs cannot be separated from EV effects on mitochondrial integrity and function, oxidative stress and inflammatory responses, which, with respect to injury, are most likely time course-dependent. Neurons rapidly reduce mitochondrial energy metabolism in the acute injury phase probably as a self-protection mechanism.^119^ The reduction of mitochondrial activity supports that dysregulated electron transport does not elicit excessive oxidative stress,^120,121^ which would otherwise exacerbate neuronal injury.^122,123^ Restorative EVs stabilize mitochondrial integrity and promote neuronal survival in the acute injury phase,^127–129^ but stimulate energy metabolism in the post-acute injury phase, when mitochondrial function is reactivated^97^ (Figure 2). The latter process boosts neuronal plasticity and neurological recovery.^97^ Hence, the stabilization of mitochondrial function, oxidative stress and pro-inflammatory responses by EVs lays the grounds for successful brain plasticity and neurological recovery.

When mitochondrial metabolism is dysregulated, neurons, their axons and synapses are prone to damage by excessive oxidative stress.^120,124,172^ Intravenously administered MSC-EVs can attenuate oxidative stress, promote neuronal survival and plasticity and enhance neurological recovery in ischemic stroke models.^27,28,83,84,86,137–139^ The recovery-promoting effects of MSC-EVs intricately involve anti-inflammatory responses in the blood and brain.^84–86,88^ That sequelae of neuronal mitochondrial dysfunction can successfully be targeted by intravenous EV application encourages clinical proof-of-concept studies in human patients. Considering the poor BBB passage of systemically administered EVs, their rapid pharmacokinetics and short circulation time, as shown in rodents^61,173–175^ and macaque monkeys,^147^ blood-derived leukocytes, which are key players in oxidative stress responses, might represent promising targets of systemically administered EVs, which transmit signals to the injured brain parenchyma.^18,85,86^ Of note, leukocytes also release their own EVs,^176^ which may amplify signals of therapeutically administered EVs. Besides leukocytes, intravenously administered MSC-EVs were shown to act on cerebral endothelial cells, inducing microvascular protection and angiogenesis.^28,87^ Cerebral endothelial cells release small EVs under conditions of ischemia, which promote neuronal plasticity and neurological recovery.^80,99^ We perhaps need to consider EVs as signal avalanches able to amplify signals on their way from the blood into the injured brain.

Besides cell sources, an important aspect in the selection of EVs relates to cell culturing conditions and EV isolation protocols. MSC-EVs, for example, may have immune tolerance-promoting or cytotoxic actions depending on the MSC culturing conditions even when a defined MSC donor is used.^85,86^ Preconditioning in the right setting by physiological or chemical stimuli may augment the restorative effects of EVs, whereas inappropriate preconditioning and loading with pro-inflammatory signals (e.g., DAMPs) or pathogenic proteins (e.g., Aβ) may abolish brain protective effects or even confer detrimental activities. When applied in ischemic stroke models, for example, hypoxic preconditioning enhanced the neurovascular, angiogenic and long-term neuroprotective effects of MSC-EVs by modifying a large number of EV proteins.^86,87^ When administered in brain cancer, the same hypoxic stimulus was found to increase tumor malignancy and growth.^177–179^ Solid pathophysiological concepts are needed with in depth knowledge about cell sources and culturing conditions to ensure that EV preparations are used that successfully stimulate neurological recovery.

A fascinating insight of experimental studies from the last years is that EVs can incorporate mitochondrial enzymes and proteins, respiration chain machineries, mitochondrial fragments or even entire mitochondria and transfer them to target cells, where they support oxidative phosphorylation and may help confine cell damage.^38,39,41^ Future studies will have to explore, to which degree mitochondrial transfer will allow us to enhance neurological recovery in different brain disease models. Various authors contributing to this special issue have provided relevant advances in this field, which might promote clinical developments.

Current challenges of the EV field are that several aspects at the intersection of the two trajectories, neuronal plasticity and mitochondrial function, are not fully characterized under pathophysiological conditions at this moment. An example is the role of neuronal EVs in coordinating presynaptic and postsynaptic plasticity, a rather recent finding.^89,90,93^ Signaling roles of EVs are strongly context-dependent.^18,20,22^ We will have to better define how endogenous EV communication contributes to the maintenance of neuronal integrity and function in different types of brain injury. Such knowledge may pave the way for novel therapeutic applications. Existing information on EVs is rapidly expanding in various brain disease areas, as exemplified by a variety of papers in this special issue.

An important message of this review are the multiple actions, via which EVs can stabilize neuronal mitochondrial function, cell survival and plasticity. Energy demands are high in the latter processes, which explains why mitochondrial integrity and neuronal plasticity are tightly linked. Having a clear therapeutic potential in a variety of brain diseases that are supported by a large number of experimental studies, the clinical translation of EV-based therapies is promising. First clinical proof-of-concept studies are on the way. We need to rule out that critical mistakes are made at this stage.

## Data Availability

The data that support the statements of this study are openly available via https://pubmed.ncbi.nlm.nih.gov/.
